# Prevalence of refractive error in Europe: the European Eye Epidemiology (E^3^) Consortium

**DOI:** 10.1007/s10654-015-0010-0

**Published:** 2015-03-18

**Authors:** Katie M. Williams, Virginie J. M. Verhoeven, Phillippa Cumberland, Geir Bertelsen, Christian Wolfram, Gabriëlle H. S. Buitendijk, Albert Hofman, Cornelia M. van Duijn, Johannes R. Vingerling, Robert W. A. M. Kuijpers, René Höhn, Alireza Mirshahi, Anthony P. Khawaja, Robert N. Luben, Maja Gran Erke, Therese von Hanno, Omar Mahroo, Ruth Hogg, Christian Gieger, Audrey Cougnard-Grégoire, Eleftherios Anastasopoulos, Alain Bron, Jean-François Dartigues, Jean-François Korobelnik, Catherine Creuzot-Garcher, Fotis Topouzis, Cécile Delcourt, Jugnoo Rahi, Thomas Meitinger, Astrid Fletcher, Paul J. Foster, Norbert Pfeiffer, Caroline C. W. Klaver, Christopher J. Hammond

**Affiliations:** 1 Department of Ophthalmology, King’s College London, St Thomas’ Hospital, London, UK; 2 Department of Twin Research and Genetic Epidemiology, King’s College London, St Thomas’ Hospital, London, UK; 3 Department of Ophthalmology, Erasmus Medical Center, Rotterdam, The Netherlands; 4 Department of Epidemiology, Erasmus Medical Center, Rotterdam, The Netherlands; 5 UCL Institute of Child Health, London, UK; 6 Department of Ophthalmology, University Hospital of North Norway, Tromsø, Norway; 7 Department of Community Medicine, UiT The Arctic University of Norway, Tromsø, Norway; 8 Department of Ophthalmology, University Medical Center, Mainz, Germany; 9 Department of Public Health and Primary Care, Institute of Public Health, University of Cambridge School of Clinical Medicine, Cambridge, UK; 10 Department of Ophthalmology, Nordland Hospital, Bodo, Norway; 11 Department of Clinical Medicine, UiT The Arctic University of Norway, Tromsø, Norway; 12 Queen’s University Belfast, Belfast, UK; 13 Research Unit of Molecular Epidemiology, Institute of Epidemiology II and Institute of Genetic Epidemiology, Helmholtz Center, Munich, Germany; 14 Univ. Bordeaux, 33000 Bordeaux, France; 15 INSERM, ISPED, Centre INSERM U897-Epidemiologie-Biostatistique, 33000 Bordeaux, France; 16 Department of Ophthalmology, Aristotle University of Thessaloniki, Thessaloníki, Greece; 17 Department of Ophthalmology, Eye and Nutrition Research Group UMR 1324 INRA, Univerisity Hospital Dijon, Dijon, France; 18 NIHR Biomedical Research Centre, Moorfields Eye Hospital NHS Foundation Trust and UCL Institute of Ophthalmology, London, UK; 19 Institute of Human Genetics, Helmholtz Center, Munich, Germany; 20 Institute of Human Genetics, Klinikum Rechts der Isar, Technische Universität, Munich, Germany; 21 London School of Hygiene and Tropical Medicine, London, UK

**Keywords:** Refractive error, Myopia, Epidemiology, Prevalence, Consortium

## Abstract

To estimate the prevalence of refractive error in adults across Europe. Refractive data (mean spherical equivalent) collected between 1990 and 2013 from fifteen population-based cohort and cross-sectional studies of the European Eye Epidemiology (E^3^) Consortium were combined in a random effects meta-analysis stratified by 5-year age intervals and gender. Participants were excluded if they were identified as having had cataract surgery, retinal detachment, refractive surgery or other factors that might influence refraction. Estimates of refractive error prevalence were obtained including the following classifications: myopia ≤−0.75 diopters (D), high myopia ≤−6D, hyperopia ≥1D and astigmatism ≥1D. Meta-analysis of refractive error was performed for 61,946 individuals from fifteen studies with median age ranging from 44 to 81 and minimal ethnic variation (98 % European ancestry). The age-standardised prevalences (using the 2010 European Standard Population, limited to those ≥25 and <90 years old) were: myopia 30.6 % [95 % confidence interval (CI) 30.4–30.9], high myopia 2.7 % (95 % CI 2.69–2.73), hyperopia 25.2 % (95 % CI 25.0–25.4) and astigmatism 23.9 % (95 % CI 23.7–24.1). Age-specific estimates revealed a high prevalence of myopia in younger participants [47.2 % (CI 41.8–52.5) in 25–29 years-olds]. Refractive error affects just over a half of European adults. The greatest burden of refractive error is due to myopia, with high prevalence rates in young adults. Using the 2010 European population estimates, we estimate there are 227.2 million people with myopia across Europe.

**Electronic supplementary material:**

The online version of this article (doi:10.1007/s10654-015-0010-0) contains supplementary material, which is available to authorized users.

## Introduction

Refractive error occurs when there is failure of the eye to correctly focus rays of light from an object onto the retinal plane. The resultant image perceived by the individual is blurred and refractive correction is required in order to see clearly. Refractive error can be divided into myopia (‘short or near-sightedness’), hyperopia (‘long or far-sightedness’) and astigmatism. In myopia, light is focussed to a point anterior to the retina as a result of excessive refraction at the cornea or lens, or, more commonly, an increased length of the eye (‘axial myopia’). In hyperopia, the reverse occurs with an image forming posterior to the retinal plane as a result of either inadequate refraction or a short axial length. In astigmatism, the refractive power of the eye is uneven across different meridians.

Refractive error requires detection and treatment in the form of glasses, contact lenses or, more recently, refractive surgery. These clinical services are readily available in most European countries, although they come with significant financial implications to both national health care systems and to individuals [1]. However, uncorrected refractive errors are still responsible for up to 42 % of the cases of visual impairment worldwide [2], and remain prevalent even in high income countries [3–6]. Uncorrected refractive error in both low and high-income countries has significant economic implications in terms of potential lost productivity [7].

The magnitude of refractive error in developed countries within individuals of European descent has been estimated by the Eye Diseases Prevalence Research Group, 10 years ago, and the US National Health and Nutrition Examination Survey (NHANES) data [3, 8]. However, the estimate of refractive error burden in Europe was based on a single cohort [9]. The European Eye Epidemiology (E^3^) consortium is a collaborative initiative between thirty-three cohort studies across Europe, to share and meta-analyse epidemiological data on eye disease in adults. The aim of the current study was to provide more current and precise estimates of the prevalence of refractive error across Europe.

## Materials and methods

### Studies and participants

To date, E^3^ has data from thirty-three studies with a range of ophthalmic data on approximately 124,000 individuals from population-based and case–control studies. This study drew on the fifteen E^3^ population-based cohort and cross-sectional studies that collected refractive error data (n = 68,350). As described in Table 1, participants included in this meta-analysis were largely from Northern and Western Europe, mainly of middle to late age, and refractive error measurements were performed between 1990 and 2013. Three studies recruited participants nationally and the remaining twelve recruited from a local population. Further detail on individual study design and sampling method is provided in the supplementary information; broadly, the majority of study samples were obtained by identification of potential participants (within defined age bands and/or regions) using local registries, with some studies using random sampling (n = 3). All studies adhered to the tenets of the Declaration of Helsinki, and relevant local ethical committee approvals with specific study consent were obtained.

**Table 1:** Description of the 15 European Eye Epidemiology consortium studies included in this meta-analysis of refractive error

Study	Data collection period	Study design	Total with refraction	Refraction method	Exclusions (cataract surgery)	Total included	Median age, years (range)	Gender, % female	Ethnicity, % European (% Unknown)	Crude myopia prevalence, %	Crude hyperopia prevalence, %
Northern Europe											
1958 British birth cohort, UK	2002–2003	Population-based birth cohort (N)	2502	Autorefraction	7 (0)	2495	44 (44–46)	51.7	98.0 (9.2)	48.7	8.8
EPIC-Norfolk, UK	2004–2011	Population-based cross-sectional study (L)	8508	Autorefraction	1110 (971)	7444	67 (48–92)	54.5	99.7 (0)	23.0	39.4
Tromsø eye study, Norway	2007–2008	Population-based cohort (L)	6565	Autorefraction	773 (700)	5792	61 (38–87)	55.9	NA (100)	19.4	33.7
TwinsUK, UK	1998–2010	National twin cohort (N)	6245	Autorefraction	161 (61)	6095	55 (16–85)	91.2	98.2 (23.9)	31.4	26.0
Southern Europe											
Thessaloniki eye study, Greece	1999–2005	Cross-sectional population-based study (L)	2259	Subjective	316 (303)	1952	69 (60–94)	44.7	100 (0)	14.2	39.4
Western Europe											
ALIENOR, France	2006–2008	Population-based cohort (L)	951	Autorefraction	333 (318)	618	79 (73–93)	56.6	NA (100)	16.7	53.6
ERF, Netherlands	2002–2005	Family-based cross-sectional study (L)	2708	Subjective	46 (45)	2662	49 (14–87)	55.1	100 (0)	21.2	27.4
Gutenberg health study, Germany	2007–2012	Population-based cohort (L)	14,679	Autorefraction	610 (610)	14,069	54 (35–74)	49.4	NA (100)	31.9	23.9
KORA, Germany	2004–2005	Population-based cohort (L)	3078	Autorefraction	706 (177)	2372	55 (35–84)	50.4	100 (0)	36.1	24.0
Montrachet, France	2009–2013	Population-based cohort (L)	1143	Autorefraction	584 (562)	576	81 (76–92)	57.5	NA (100)	19.1	51.1
Rotterdam Study I, Netherlands	1990–1993	Population-based cohort (L)	6748	Subjective	182 (172)	6566	68 (55–106)	59.3	98.5 (2.0)	16.4	52.3
Rotterdam Study II, Netherlands	2000–2002	Population-based cohort (L)	2689	Subjective	110 (110)	2579	62 (55–99)	54.8	87.8 (0.1)	21.9	45.7
Rotterdam Study III, Netherlands	2005–2008	Population-based cohort (L)	3624	Subjective	94 (74)	3530	56 (46–97)	56.3	NA (100)	32.5	28.8
POLA, France	1995–1997	Population-based cohort (L)	2464	Autorefraction	157 (128)	2315	70 (60–93)	55.8	NA (100)	16.2	53.0
Mixed											
EUREYE: Norway, UK, France, Italy, Greece and Estonia	2000–2002	Population based cross-sectional survey in seven cities (L)	4187	Autorefraction or focimetry with subjective refraction	1305 (517)	2882	72 (65–95)	56.7	NA (100)	15.6	59.2
Total cohort	1990–2013		68,350		6404 (4748)	61,946	62	57.6	98.1	25.8	34.4

Myopia ≤−0.75 diopters (D), hyperopia ≥1D, *N* national, *L* local

### Inclusion and exclusion criteria

Studies in the E^3^ consortium were eligible for inclusion in this analysis if they were population-based, and data on refraction, together with age at measurement and year of birth, were available. Study participants were excluded if they were identified as having had cataract surgery, retinal detachment, refractive surgery or other factors that might influence refraction (e.g. keratoconus), at the discretion of each study’s analysis team.

### Demographic and outcome variables

All included studies measured non-cycloplegic refraction (i.e. no dilating drops were used) using the technique of subjective refraction, autorefraction or a combination of focimetry (measuring an individuals glasses) or autorefraction followed by subjective refraction (Table 1). Participant’s spherical equivalent (SE) was considered as the mean SE of the two eyes calculated using the standard formula (SE = sphere + (cylinder/2)). Refractive error was categorized using the following definitions: myopia ≤−0.75 diopters (D), low myopia ≤−0.75 to >−3D, moderate myopia ≤−3D to >−6D, high myopia ≤−6D, hyperopia ≥1D, high hyperopia ≥3D and astigmatism ≥1D. Definitions of myopia vary in the literature; the cut-off of −0.75D was chosen as unaided visual acuity at this level approximates 0.3 LogMAR (Logarithm of the Minimum Angle of Resolution) [10], a commonly used driving standard, and this has been used in recent international meta-analyses of the genetic epidemiology of refractive error and myopia [11].

Differences in age (in 5 year age bands from ≥15 to ≥90 years), gender (male/female) and geographical European region were examined. Geographical variations in the prevalence of myopia were investigated by dividing countries in three areas (Northern, Western and Southern Europe) according to the United Nations Geoscheme [12]. Information on ethnicity, when available, was recorded using a modified classification system based on genetic ancestry [13].

### Statistical analysis

Study specific summary data were obtained. A random effects meta-analysis was performed for spherical equivalent and repeated for refractive classifications overall and stratified by age. This enabled calculation of pooled estimates of refractive error prevalence, with studies weighted by sample size and between-study variance and a summary estimate standard error calculated from the inverse sum of the adjusted weights. A random effects model was chosen over a fixed effects model, to allow for heterogeneity in study design characteristics.

Age-standardised prevalences were calculated using the following steps: firstly, age-specific prevalences were estimated using random-effect meta-analyses. Secondly, an age-standardisation with adjustments to age-specific estimates according to the European Standard Population 2010 was performed [14]. This enabled refractive error prevalence estimates that are representative for the European population, with appropriate weighting to the age demographic distribution of Europe.

Subsequent random effects meta-analyses were performed with stratification by age and gender, and subsequently age and geographical region, with differences between groups evaluated using ANOVA tests.

Statistical analysis was performed using Stata version 13.1 (StataCorp. 2013. Stata Statistical Software: Release 13. College Station, TX: StataCorp LP). Graphical outputs were obtained using either Stata or ggplot2 [15] in R (R Core Team (2014). R: A language and environment for statistical computing. R Foundation for Statistical Computing, Vienna, Austria. URL http://www.R-project.org).

## Results

**Fig. 1:**
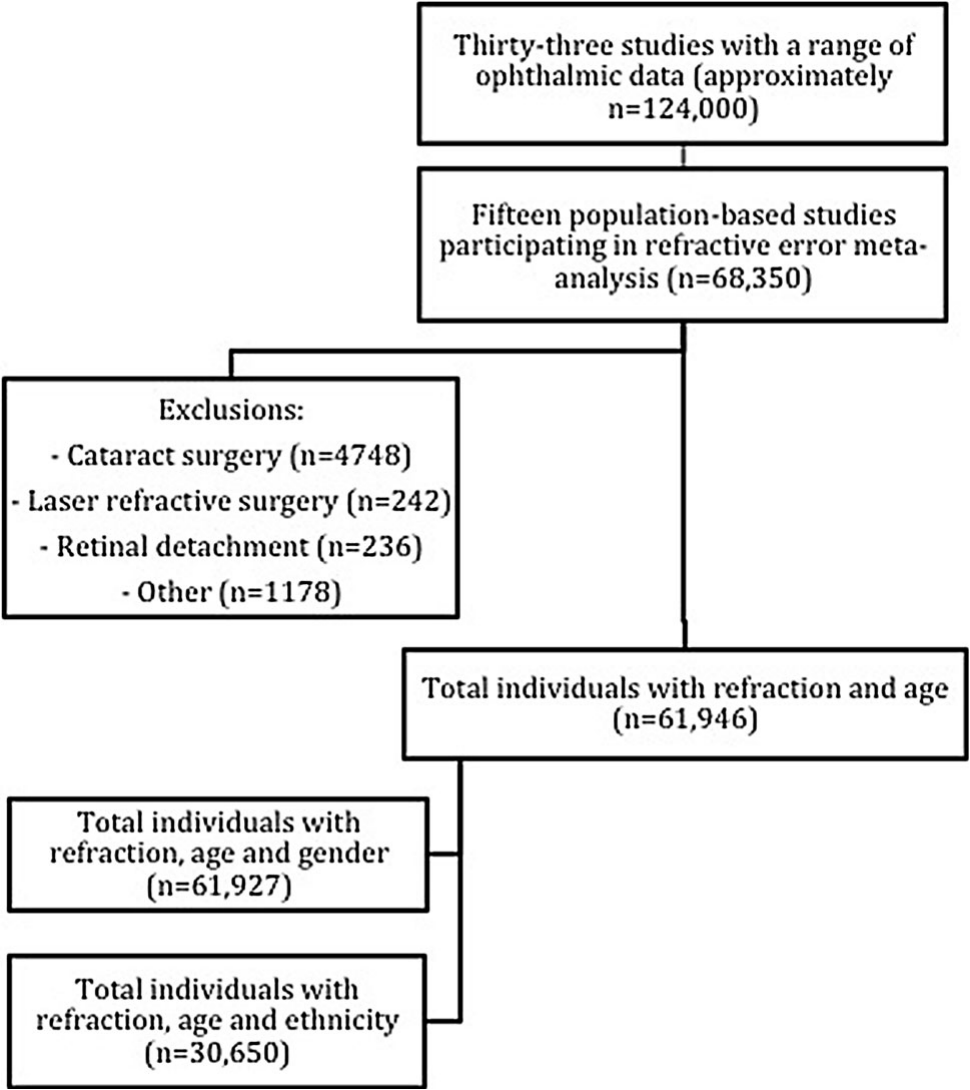
Flow chart of refractive error meta-analysis within E^3^

Fifteen studies contributed a total of 61,946 individuals after exclusions (Fig. 1). The median age of the included populations ranged from 44 to 78 years old (Table 1). There was a slight female predominance in the combined study (57.6 % females). Data on ethnicity was only available for 50 % of participants, and in these there was minimal ethnic diversity (98 % European ancestry), so no further analysis of ethnicity was carried out.

**Fig. 2:**
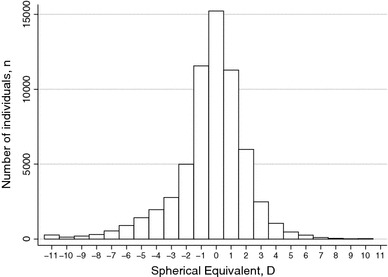
Distribution of refractive error (*D* diopters)

The distribution of refractive error displayed a leptokurtotic distribution (Fig. 2), with a median spherical equivalent of 0.56D (range −25.13–22.19). The distribution was asymmetric with a greater frequency of individuals with a negative refractive error.

**Fig. 3:**
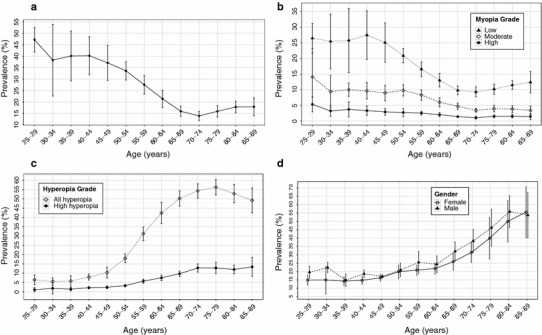
**a** Prevalence of myopia (SE ≤−0.75D) according to age, with 95 % confidence intervals (*D* diopters). **b** Prevalence of myopia (low myopia SE ≤−0.75 to >−3D, moderate myopia SE ≤−3 to >−6D, high myopia SE ≤−6D) according to age, with 95 % confidence intervals (D, diopters). **c** Prevalence of hyperopia (all hyperopia SE ≥1D, high hyperopia SE ≥3D), according to age, with 95 % confidence intervals (*D* diopters). **d** Prevalence of astigmatism (≥1D) according to age for males and females with 95 % confidence intervals (*D* diopters)

Given there were only 314 participants aged 15–24 years and 156 >90 years of age, subsequent analyses are limited to those aged ≥25 and <90 years (n = 61,476). The overall myopia prevalence in our meta-analysis was 24.2 % (95 % confidence interval (CI) CI 19.9–28.5), with a European age-standardised myopia prevalence of 30.6 % (95 % CI 30.4–30.9; Table 2). Myopia was most common in younger participants [peaking at 47.2 % (95 % CI 41.8–52.5) in those aged 25–29 years], almost double the prevalence of those of middle and older age [27.5 % (95 % CI 23.5–31.5) in those aged 55–59 years; Fig. 3a]. Point estimates of myopia prevalence in those aged 15–19 years were 27.4 % (95 % CI 17.0–37.8), increasing to 34.2 % (95 % CI 27.9–40.6) in those aged 20–24 years. All degrees of myopia followed a similar pattern of higher prevalence in the younger cohorts, lower prevalence in the middle aged and more elderly participants, and an increase in the very eldest participants, albeit with wide CIs, most likely related to cataract development. Age-standardised prevalence of high myopia across all age groups was 2.71 % (95 % CI 2.69–2.73), with 3–5 % of young to middle-aged individuals affected and 1–2 % of older individuals (Fig. 3b).

**Table 2:** Prevalence of myopia, hyperopia and astigmatism stratified by age

Age	n	Myopia, % (95 % confidence intervals)				Hyperopia, % (95 % confidence intervals)		Astigmatism, % (95 % confidence intervals)
		All myopia ≤−0.75D (n = 15,845)	Low myopia ≤−0.75 to >−3D (n = 10,034)	Moderate myopia ≤−3 to >−6D (n = 4383)	High myopia ≤−6D (n = 1445)	All hyperopia ≥+1D (n = 21,201)	High hyperopia ≥+3D (n = 4494)	All astigmatism ≥D (n = 15,496)
25–29	339	47.2 (41.8–52.5)	26.5 (21.8–31.2)	14.1 (5.1–23.2)	5.3 (2.9–7.7)	6.4 (3.8–9.0)	1.1 (0.0–2.2)	16.2 (12.3–20.1)
30–34	469	38.3 (22.6–53.9)	25.5 (16.7–34.2)	9.4 (4.2–14.6)	3.2 (1.5–4.9)	5.5 (3.4–7.5)	1.8 (−1.1–4.6)	18.2 (14.3–22.0)
35–39	2354	40.1 (29.2–51.0)	25.8 (15.5–36.0)	10.0 (7.9–12.1)	3.7 (1.3–6.1)	5.8 (3.0–8.6)	1.4 (0.5–2.3)	16.2 (14.5–17.9)
40–44	5552	40.2 (32.0–48.5)	27.5 (19.7–35.3)	9.6 (7.0–12.3)	3.3 (1.8–4.8)	7.9 (6.3–9.5)	2.2 (1.6–2.7)	15.7 (13.2–18.1)
45–49	4108	37.1 (29.4–44.7)	25.1 (18.8–31.4)	9.0 (6.5–11.4)	2.9 (1.8–4.0)	10.3 (7.5–13.2)	2.4 (1.7–3.1)	17.0 (15.1–18.8)
50–54	5684	33.6 (29.6–37.6)	20.9 (18.6–23.2)	9.8 (8.0–11.6)	2.7 (1.4–4.0)	18.0 (15.6–20.4)	3.3 (2.6–3.9)	20.1 (16.3–23.8)
55–59	8294	27.5 (23.5–31.5)	16.6 (14.2–18.9)	8.3 (6.6–9.9)	2.5 (1.9–3.1)	31.2 (27.5–34.9)	5.7 (4.6–6.8)	22.5 (18.2–26.9)
60–64	10,594	21.4 (17.5–25.2)	13.0 (10.9–15.2)	6.0 (4.5–7.4)	2.0 (1.4–2.7)	31.2 (27.5–34.9)	7.5 (6.0–9.0)	25.2 (20.3–30.0)
65–69	9445	15.9 (13.7–18.1)	9.8 (8.4–11.2)	4.7 (3.7–5.7)	1.4 (1.1–1.6)	50.2 (46.1–54.3)	9.7 (8.2–11.1)	28.0 (22.0–34.0)
70–74	7674	13.9 (11.9–15.9)	9.3 (7.8–10.9)	3.4 (2.8–4.0)	1.0 (0.6–1.5)	54.3 (50.4–58.1)	12.8 (9.9–15.7)	33.8 (26.6–41.1)
75–79	4211	15.9 (13.4–18.4)	10.2 (8.5–11.8)	3.9 (2.9–5.0)	1.5 (1.0–1.9)	56.3 (52.1–60.4)	12.8 (9.9–15.7)	44.3 (33.6–55.0)
80–84	2069	17.8 (15.2–20.3)	11.5 (10.1–12.9)	3.8 (2.7–4.9)	1.5 (1.0–2.1)	52.8 (47.9–57.7)	12.0 (9.7–14.3)	51.1 (40.4–61.8)
85–89	683	17.9 (14.0–21.8)	12.4 (9.0–15.8)	3.4 (2.0–4.8)	1.4 (0.4–2.3)	49.2 (42.5–55.9)	13.4 (8.4–18.5)	54.9 (42.9–66.8)
Age standardised prevalence (n = 61,476)		30.60 (30.36–30.85)	19.50 (19.35–19.65)	8.08 (8.01–8.14)	2.71 (2.69–2.73)	25.23 (25.03–25.43)	5.37 (5.33–5.41)	23.86 (23.67–24.05)

*D* diopters

Overall prevalence of hyperopia was 34.7 % (95 % CI 27.9–41.6), with an age-standardised prevalence of 25.2 % (95 % CI 25.0–25.4). There was less hyperopia in young participants [6.4 % (95 % CI 3.8–9.0) in those aged 25–29 years], compared to those in middle to older age [31.2 % (95 % CI 27.5–34.9) in those aged 55–59 years] although hyperopia rates declined after 75 years of age. The prevalence of high hyperopia followed a similar pattern, affecting 1–3 % of younger and 10–13 % of older individuals (Fig. 3c). Across all ages, the prevalence of astigmatism was 27.3 % (95 % CI 22.6–32.1) with an age-standardised estimate of 23.9 % (95 % CI 23.7–24.1). The prevalence of astigmatism remained fairly stable at 15–25 % in young and middle-aged participants [17.0 % (95 % CI 15.1–18.8) in those aged 45–49 years]. However, in participants over 65 years of age, astigmatism became more common [51.1 % (95 % CI 40.4–61.8) in those aged 80–84 years; Fig. 3d].

Age- and gender- specific analyses for myopia, hyperopia and astigmatism are reported in Table 3. There were no significant differences in myopia prevalence between men and women across age strata. However, overall there was a significantly higher prevalence of astigmatism in men (*p* = 0.001), with a mean difference of 3.8 % across all ages, and a significantly higher prevalence of hyperopia in women (*p* = 0.04) with a mean difference of 2.5 % across all ages.

**Table 3:** Prevalence of myopia, hyperopia and astigmatism stratified by age and gender

Age	n		Myopia, ≤−0.75D (95 % confidence intervals)		Hyperopia, ≥+1D (95 % confidence intervals)		Astigmatism, ≥1D (95 % confidence intervals)	
	Women	Men	Women	Men	Women	Men	Women	Men
25–29	278	59	47.9 (40.0–55.8)	40.2 (22.7–57.8)	6.1 (6.1–6.2)	11.2 (−1.5–23.8)	14.9 (12.0–17.7)	19.6 (15.9–23.3)
30–34	307	160	40.3 (32.6–47.9)	41.7 (10.7–72.7)	4.3 (0.0–8.6)	5.2 (2.9–7.5)	15.0 (6.7–23.2)	22.4 (19.4–25.5)
35–39	1352	1004	40.2 (30.5–49.8)	40.9 (26.2–55.5)	5.6 (3.0–8.1)	6.5 (3.8–9.2)	14.3 (11.7–16.9)	14.9 (11.1–18.7)
40–44	2989	2561	39.8 (31.4–48.2)	42.0 (33.4–50.6)	7.9 (6.6–9.1)	8.4 (7.0–9.8)	14.8 (12.5–17.1)	18.7 (16.3–21.0)
45–49	2258	1849	37.1 (29.8–44.3)	37.1 (25.8–48.3)	11.4 (9.1–13.8)	8.3 (6.6–9.9)	16.3 (14.4–18.2)	17.1 (16.0–18.2)
50–54	3369	2315	34.1 (30.0–38.3)	33.0 (27.7–38.3)	19.1 (17.6–20.7)	17.3 (15.1–19.5)	19.9 (15.8–23.9)	20.9 (16.9–25.0)
55–59	5086	3206	25.8 (21.6–30.1)	29.8 (23.7–35.9)	32.6 (29.8–35.5)	30.6 (25.9–35.2)	20.9 (17.8–23.9)	25.5 (18.8–32.1)
60–64	6364	4226	19.1 (15.9–22.3)	20.7 (17.0–24.4)	43.7 (38.8–48.6)	36.1 (31.9–40.2)	21.8 (18.5–25.1)	24.4 (19.4–29.4)
65–69	5207	4237	14.5 (12.0–17.1)	16.6 (14.2–18.9)	52.3 (48.2–56.4)	48.4 (44.7–52.1)	25.9 (20.9–30.9)	32.1 (26.3–37.8)
70–74	4110	3562	14.2 (12.2–16.2)	14.3 (12.3–16.3)	55.8 (52.2–59.4)	55.0 (51.5–58.5)	31.4 (25.5–37.3)	38.3 (31.5–45.1)
75–79	2290	1920	14.3 (11.5–17.0)	17.7 (14.9–20.5)	58.0 (55.3–60.6)	52.3 (48.0–56.7)	39.9 (35.1–57.2)	46.2 (35.1–57.2)
80–84	1158	911	15.8 (12.8–18.7)	18.7 (16.2–21.3)	57.6 (53.0–62.2)	47.8 (42.6–53.0)	50.0 (37.6–62.4)	55.8 (46.3–65.3)
85–89	419	264	19.6 (13.2–25.9)	16.1 (11.7–20.5)	50.7 (43.5–57.9)	45.0 (37.9–52.0)	55.5 (40.3–70.6)	53.8 (40.2–67.3)
*p* diff between groups (ANOVA)			0.603		0.042		0.001	

*D* diopters

Differences in the myopia prevalence between different European regions, according to the UN European Geoscheme, were examined. Only one cohort contributed to the Southern European division (Thessaloniki Eye Study, Greece), with participants all over the age of 60 years, thus the majority of the studies were in Northern and Western regions. The prevalence of myopia did not differ between Northern and Western countries and followed a similar pattern across all age groups. The single Southern participant cohort appeared to have a higher level of myopia in its older participants when compared to Northern and Western countries, however there were large CIs for these estimates (80–84 year-old myopia prevalence in North 13.6 % (95 % CI 9.3–18.0), West 18.0 % (95 % CI 16.1–21.1) and South 29.1 % (95 % CI 19.1–39.1). Overall there were no significant differences across age strata between the three regions of Europe studied (*p* = 0.70).

## Discussion

Meta-analysed data from fifteen population-based adult cohort and cross-sectional studies across Europe indicated age-standardised prevalence of 30.6 % for myopia, 25.2 % for hyperopia and 23.9 % for astigmatism. This meta-analysis usefully incorporates data from across Europe and is not limited to a particular place or age group. The most significant burden of refractive error within Europe was from myopia.

A clear trend of higher levels of myopia in younger individuals was identified, with a rising prevalence during late teens and 20 s reflecting the known natural history of the condition [16]. The peak prevalence of myopia was identified in the 25–29 years age group (47.2 % (95 % 41.8–52.5). In older individuals, the prevalence of myopia was lower, for example 15.9 % (95 % CI 13.7–18.1) in those aged 65–69 years old. This may reflect the rising prevalence of myopia in younger generations, or the known hyperopic shift in aging [17, 18]. In our aged 75 or over participants, there was an increase in myopia prevalence. While we aimed to exclude those having undergone cataract surgery (and participants with documented cataract in some studies), the rise in myopia likely reflects the development of nuclear cataract, which is known to be associated with a myopic shift as a result of increasing lens power [19]. However, this age-related change in refraction may also occur irrespective of visible lens opacity; in the Beaver Dam Study, a 10-year longitudinal myopic shift (−0.19D, 95 % CI −0.32 to −0.06, *p* < 0.001) was observed in those over 70 years old, even after adjusting for nuclear sclerosis grading [17]. We did not confirm the observation of previous studies of higher myopia prevalence in women [20].

In comparison to previous estimates, the overall burden of myopia in our population appears similar but slightly greater to that of other studies. The 2004 Eye Diseases Prevalence Research Group estimated myopia prevalence at 26.6, 25.4 and 16.4 % for European, North American and Australian sub-analyses respectively [8]. This study included the Beaver Dam Eye Study [21], the Baltimore Eye Survey [22], the Blue Mountains Eye Study [23], the Melbourne Visual Impairment Project [24] and the Rotterdam Study I [9], which was also included in this meta-analysis. In their youngest cohort (40–49 years), 36.8 % of white men and 46.3 % of white women were myopic, similar to our estimates of 42.0 and 39.8 % in 40–44 year-olds, albeit with no gender difference. The US 1999–2004 NHANES examined refractive error variation by age in three ethnicities; the prevalence of myopia in non-Hispanic white participants 20–39 years of age was 35.1 % in men and 42.3 % in women, whilst the prevalence in those ≥60 years was 23.1 % in men and 18.6 % in women [20]. These prevalence rates are again very similar to that found in our data, although we did not find higher levels of myopia in young females. Both comparative estimates are based on a definition of myopia ≤−1D, and are therefore not directly comparable to our study definition of myopia ≤−0.75D, an issue often encountered in refractive error epidemiology where there is a lack of consensus on definitions of refractive error. The adult prevalence of myopia in South-east Asia is of much greater magnitude than that seen in studies of European ancestry [25–28], with remarkably high levels of myopia seen in young individuals [29, 30]. The number of participants in our meta-analysis of Asian origin was very low, precluding meaningful reporting of these estimates.

High myopia prevalence was relatively low in Europe, with an age-standardised estimate of 2.7 % (95 % CI 2.69–2.73). The highest prevalence was observed in younger participants, albeit with wider CIs due to smaller sample size (Table 2). Prevalence in older participants was low, potentially reflective of generational changes, or perhaps exclusion due to the earlier need for cataract surgery in high myopes compared to other refractive groups [31]. Our greatest high myopia prevalence of 5.9 % (95 % CI 1.3–10.5) in 15–19 year-olds remains much lower than that seen in, for example, urban China where up to 14 % of 17 year-olds are highly myopic [32]. In non-Hispanic White individuals in the NHANES 1999–2004 data, high myopia appeared slightly more common than in our data; for example in those aged 20–29 years-old “severe” myopia was identified in 7.4 %, compared to 2.8 and 5.3 % in those aged 20–24 and 25–29 respectively in this European study. However the NHANES definition of severe myopia (≤−5D) again differs slightly from our definition of high myopia (≤−6D).

Using the same definition of high hyperopia (≥ 3D), our study appeared to have less hyperopia than the Eye Diseases Research Group [8]; for example in 70–74 year-olds 21.3 % of white women and 16.9 % of white men were highly hyperopic compared to just 12.8 % in our European data, which may again reflect a generational or cohort effect.

Astigmatism rates were fairly constant (15–25 %) across cross-sectional age categories, but were higher after the age of 65. This finding has been observed in other studies, together with a shift from with-the-rule to against-the-rule astigmatism [20, 23, 28]. Across all age groups, we identified higher astigmatism prevalence in men, particularly evident in middle to later ages (for example 39.5 % in women and 46.2 % in men aged 70–74). This observation was similar in the older participants of the NHANES 1999–2004 study, where in participants over the age of 60 years the astigmatism prevalence in women was 46.1 % and in men 54.9 % [20].

The major strength of our study is the large sample size contributing to the prevalence estimates, providing a unique opportunity to estimate the burden of refractive error in middle and older aged individuals across Europe. This is beneficial for planning of clinical services and raises awareness, for both clinicians and economists, of the future potential issues of rising myopia levels and associated visual impairment [33]. Refractions were all non-cycloplegic, which is common practice for population-based adult ophthalmic epidemiological studies, thus making this study comparable to previous research [34, 35].

Despite age and gender stratification, significant heterogeneity between studies remained in the meta-analysis. There are inherent differences in the included studies in terms of study design, refraction technique and cohort sampling, together with between country differences in levels of urbanisation, economy, education and climate which may influence refractive error. We were unable to stratify by these factors in this meta-analysis as person-specific data was not available for all studies. This study was mainly comprised of middle and older aged individuals, therefore our estimates of refractive error prevalence carry greater confidence for these ages since they are based on more precise estimates with narrow 95 % CIs. The majority of the studies in this meta-analysis originate from Northern and Western European countries, and therefore our estimates of refractive error are more representative of these European countries. Although our sample includes either national or locally recruited population-based studies, like all epidemiological studies there may be a bias of participants volunteering for an eye examination being more ‘health conscious’. We suspect this would have little effect on the prevalence of refractive error, and if anything result a slight underestimation of the prevalence. Finally, refractions were performed over a twenty-year period and, therefore our estimates of prevalence may be subject to error given temporal trends in refractive error prevalence. However, refractions were performed between 2000 and 2010 in thirteen out of the fifteen studies, reducing this variability.

In conclusion, this study estimates refractive error affects just over a half of European adults. Myopia represented the greatest burden, with an estimated 227.2 million people across Europe affected (using the 2010 European population estimates) [36]. Based on study prevalence estimates of high myopia, this also suggests there are 20.1 million people across Europe who are at higher risk of the associated sight threatening complications, such as retinal detachment, that this degree of myopia confers [33].

## Electronic supplementary material

Below is the link to the electronic supplementary material.

Supplementary material 1 (DOCX 61 kb)
